# Chairman’s Address, Section of State Medicine and Public Hygiene*Read in the Section of State Medicine at the Thirty-fourth Annual meeting of the State Medical Association of Texas, at Dallas, Texas, May 8, 1901, and by resolution ordered printed in pamphlet form for distribution for “educational purposes.”

**Published:** 1902-06

**Authors:** Wm. S. Carter

**Affiliations:** Galveston, Texas


### Chairman's Address, Section of State Medicine and Public Hygiene.*

BY WM. S. CARTER, M. D., GALVESTON, TEXAS.

In assuming the duties of chairman of this section, I beg to express my appreciation of the honor conferred by appointment to the position, and at the same time to congratulate the Association on the wisdom of maintaining a separate Section on State Medicine and Public Hygiene. At a time when the program is crowded with papers on subjects which are often regarded as more practical than hygiene, it might seem to some, inadvisable to continue such a separate section.

Preventive medicine, however, is not an impractical subject, as some physicians seem to regard it, but one which is eminently practical. Although its development has been of comparatively recent date, its achievements up to the present time and the possibilities for the future are not exceeded by those of any other branch of medicine.

It has not been very long since the study of hygiene was regarded largely as a consideration of personal hygiene, the composition and

---

*Read in the Section of State Medicine at the Thirty-fourth Annual meeting of the State Medical Association of Texas, at Dallas, Texas, May 8, 1901, and by resolution ordered printed in pamphlet form for distribution for "educational purposes."

adulteration of foods, the air, water, soil, etc. While attention to these matters promotes health and happiness, yet the fact remains that, with the single exception of smallpox, we had no rational, scientific basis for the prevention of any of that great group of diseases which we call infectious and contagious before the advent of bacteriology. Along with the rapid development of this new science, during a little more than two decades, have come the greatest strides in preventive medicine. Most of these advances have resulted from a knowledge of the specific cause of the different contagious diseases. Today sound preventive measures are not limited to smallpox alone, but are applied to cholera, typhoid fever, plague, sepsis in its various forms, tuberculosis, diphtheria and (happily for us living in Southern States) to malaria and yellow fever. Probably no advance of modern medicine has been hailed with more delight by physicians of Southern States than the discoveries of the manner in which yellow fever and malaria are spread and the certain methods by which they can be prevented.

Malaria is so wide-spread and has caused so much suffering that notwithstanding the fact that we possess a specific remedy for the disease, we are led to hope that preventive measures may ultimately be productive of more good than the curative. The practical results of prophylactic measures against malaria may not be so striking as those obtained with other preventable diseases, but it should be remembered that it is impossible to represent in figures the number of infections with this disease that may be prevented. We should also bear in mind that with the greatest care it was impossible to prevent infection with malaria before it was found that the mosquito carries the infection. On the other hand, the fact that people escape the disease when living in malarial districts under exactly the same condition as others, except that they protect themselves against mosquitoes, shows what can be accomplished by rigid preventive measures.

With yellow fever results are more immediate and decisive. The brilliant investigation of the Medical Board of the U. S. Army,[1] Drs. Reed, Carroll, Agramonte, and the much lamented Lazear, are as epoch-making in the case of yellow fever as were those of Jenner in dealing with smallpox. The results obtained by these gentlemen have been fully confirmed by the more recent observations of Dr. John Guiteras.[2]

They have not only demonstrated that the disease is spread by the mosquito, but that it is probably the *only way* in which it is spread. It is now easy to understand certain well-known facts about the spread of this disease, such as the peculiar house infection; the influence of cold in stopping epidemics: the failure of the disease to spread in localities at higher altitudes, even when numerous cases are introduced from centres of infection, etc.

Never before have the results of a scientific investigation into the spread of a communicable disease been so promptly and efficiently applied toward suppressing and stamping out that disease as in this instance. From 1853 to 1900 the average yearly mortality from yellow fever in the city of Havana was 765; for the past eleven years (1888 to 1899), it was 440. From April 1, 1901, to January 1, 1902, there were five deaths, and two of these were volunteers who submitted to experimental inoculation with mosquitoes.

Dr. Gorgas, Surgeon and Major, U. S. A., Chief Sanitary Officer of Havana, in his report for December, 1901, said. "Taking the months of October, November and December from 1890 to 1900, inclusive, the average number of deaths for those months was 144.5, the largest number being 631.

"In 1901 for the same period we had no deaths. This is the only year in which any attention has been paid to mosquitoes in connection with yellow fever. In the two preceding years of American occupation (1899 and 1900) every means known to science and which money could command, backed by unlimited military authority, was used to destroy fomites on the theory that the fomites were the means of propagating yellow fever. In 1901 the same efforts were directed to the destruction of mosquitoes on the theory that the mosquito is the only means of propagating this disease, the effort to destroy fomites having been entirely laid aside."

The results are well expressed by Guiteras,[3] who says, "The complete control over the spread of yellow fever that the Sanitary Department of Havana has obtained this year by the enforcement of prophylactic measures that are based solely on the doctrine of the transmission of yellow fever by the mosquito, goes very far to prove that there is no other channel of communication of the disease. A few sporadic cases have occurred; some have been imported from the interior; but in every instance the propagation of the disease has been arrested. At the present writing (September 13, 1901), there is but one case of yellow fever in the City of Havana. At this time the annual epidemic is always at its height. I do not know of a greater victory in the history of sanitary science. Had the American intervention in Cuba done nothing else for humanity, it might well stand upon that record and call upon the coming years of the century to surpass it."

While we rejoice in the fact that epidemic diseases like cholera and yellow fever are now robbed of their terrors, and point with pardonable pride to the tremendous reduction in the mortality rates from such a disease as diphtheria, which was formerly beyond our control, it is well for us to pause and inquire if prophylactic measures are generally carried out by physicians in private practice in the management of preventable diseases.

During the first two months of the present year there were officially reported for the United States 20,044 cases of smallpox, with 615 deaths, as compared with 7,637 cases with 104 deaths for the corresponding period in 1901. This is indeed a sad comment on our methods of dealing with a disease which we boast of as being preventable!

With optional vaccination, our indifference to preventable diseases in general, and the lack of uniformity in our sanitary laws, we are too prone to neglect vaccination as a routine prophylactic measure and only carry it out spasmodically and often incompletely after an epidemic of smallpox starts. Our regard for the liberties of the individual causes us to do a great injustice to a large number of people by allowing anti-vaccination cranks, usually by means of false statements, to prejudice people who are incapable of judging for themselves of the merits of vaccination.

Contrast with the numbers just given above, the results of compulsory vaccination. In Germany, since the vaccination of infants has bcome cumpulsory, with revaccination at school age, the death rate has been reduced to seven per million, as compared with 309 per million under the laws of optional vaccination. In the German army, where vaccination is most rigidly enforced, there has been but one death from smallpox since 1874.

According to the vital statistics of the census returns, there were 35,379 deaths from typhoid fever in 1900, while the number in 1890 was 27,058. This increase is out of proportion to the increase of population, notwithstanding the great reduction in mortality rates which has followed the more general use of cold water in the treatment of this disease. What is the cause of this prevalence of typhoid fever? You may answer that it arises from conditions attending the rapid growth of this great country and which results in the pollution of the water supplies to large cities as well as small towns; that the spread of this disease by insects has been overlooked until quite recently; also that many mild cases are not treated. We will grant all this, but back of it all, are the general medical practitioners—the physicians who attend cases of typhoid fever—doing their whole duty? Do they see that the discharges by which the disease is communicated from the sick to the well are properly disinfected in each case they attend? If family physicians were doing their entire duty in this regard, would the disease be on the increase? If we look at this matter squarely, I fear we will be obliged to answer in the negative. Physicians in private practice are too prone to overlook the duty they owe to the well in their concern and attention to the sick. While our first duty is to the sick, we should not neglect prophylactic measures to prevent the spread of disease to the well.

There is no disease in which preventive measures are more commonly neglected than in the case of tuberculosis, and there is no disease in which there is greater need of care in this direction. The United States Census Report for 1900 states that there were 111,000 deaths from tuberculosis for that year. This, however, does not represent the total number, as only one-third of the entire population is included in that registration area, *i. e.,* States and cities having reliable mortality records. Taking the mortality rate (190.5) per 100,000 in this area and applying it to the entire population, it is found that there were 145,000 deaths from tuberculosis in this country for 1900, as compared with 154,000 for 1890. It might be contended that this estimate is too high, as the registration area includes mostly the urban population, while the non-registration area represents largely the rural districts. Against this argument it may be stated that the total number of deaths recorded from all causes for the non-registration area was 526,425, although the population was nearly two-thirds that of the entire country (over 47 millions), while the total number of deaths from all causes in the registration area was 512,669. The latter only represents a little over one-third (28 millions) of the total population. It is quite evident from this that the vital statistics of the non-registration districts are unreliable, and it is entirely safe to say that there were approximately 150,000 deaths from tuberculosis in the United States every year from 1890 to 1900.

We can get a beter idea of the significance of these figures by comparing them with other great losses of life. It is estimated that the total number of lives lost on both sides during the great Civil War from 1861 to 1865 amounted to three-quarters of a million. It will be seen, therefore, that the number of deaths from tuberculosis in this country between the last two census enumerations (1890-1900) was twice as great as the total number of deaths from all causes on both sides during the war between the States.

In an excellent article published last year on the prevention of yellow fever,[5] Drs. Reed and Carroll have collected the statistics of all the epidemics of yellow fever in this country from 1793 to 1900, and they find that the total number of deaths was about 100,000. In other words, the total number of deaths from yellow fever for over a century only amounted to two-thirds of the number of deaths that occur from tuberculosis in this country every year.

At the present day no one will deny the infectious nature of tuberculosis. It is equally well known that the bacillus of tuberculosis does not thrive and grow well outside the animal body. It naturally follows, then, that every case of tuberculosis is caused by the germs given off by some previous case of the same disease either in man or the lower animals. If any one denies the communicability of this disease, he must first disprove these statements.

It is difficult to understand why physicians who use reasonable prophylactic measures in dealing with diphtheria, scarlet fever, typhoid fever, etc., should not be just as careful to prevent the spread of tuberculosis. They are often entirely indifferent to the prevention of this disease, which, according to the last census reports, kills as many people as all other common communicable diseases put together, if we exclude pneumonia.

There are some who admit that the disease is infectious, but they contend that it is not very communicable. This is probably because a prolonged exposure or repeated exposures are necessary to develop the disease. No one would assert that tuberculosis is highly contagious, *i. e.,* directly contagious in the ordinary use of this term. It is probably better to use the term communicable instead of contagious in order to avoid confusion in this matter. If the disease is *not* communicable, then it remains for those who advance this argument to show that tuberculosis may develop in some other way than from the germs given off by tuberculous patients. If it *is* communicable, then we should use common sense methods for destroying the germs as soon as they are given off from every patient suffering from the disease, and thus prevent their dissemination.

Since we know pretty well how the disease is conveyed from the sick to the well, one would naturally expect that every effort would be made by physicians and by people generally to check its spread. It is surprising to see how little attention is generally paid to this matter. From the inquiries of Dr. Knopf[6] into the present status of the preventive measures against tuberculosis in the United States, we learn that only in fourteen States of the Union are there any laws to prevent the spread of bovine tuberculosis and providing for the dissemination of circulars of information concerning tuberculosis in man. As a matter of fact, systematic efforts toward the prevention of the disease are actually only carried out in a very few of these States. Dr. Knopf further addressed letters of inquiry to the health authorities of forty of our largest cities, and one-third of those replying stated that nothing whatever was being done by these municipalities toward the prevention of this disease.

Those who have followed the pioneer work done in this direction by the Board of Health of New York City will recall the antagonism and harsh criticism which were offered at first. This feeling has gradually changed as physicians and the people have come to understand the methods better, and today thoughtful men will commend rather than criticise the work of these progressive and practical sanitarians.

Others will present to this Section important facts concerning the spread and practical methods of preventing tuberculosis. I trust you will pardon me if, instead of attempting to review the year's progress in sanitary science, as is customarily done in the chairman's address, I call your attention briefly to some facts concerning the viability of the bacillus of tuberculosis outside the animal body.

### THE VIABILITY OF THE BACILLUS OF TUBERCULOSIS OUTSIDE THE ANIMAL BODY..

There is a general impression among the medical profession that the bacillus of tuberculosis loses its virulence soon after it is given off from the diseased tissue. The conditions which cause this loss of pathogenicity are, (1), the putrefaction caused by other bacteria commonly present in sputum and other tuberculous discharges; (2), the dessication of these discharges; and, (3), the influence of light, especially sunlight.

Concerning the influence of putrefaction, Baumgarten, Fischer, DeFoma and Falk[7] stated that tubercle bacilli are killed by putrefaction in from eight to ten days.

Schill and Fischer deny this and find the bacilli virulent after exposure to putrefaction for forty-three days. Koch agrees with the latter view. Cardeac and Malet[9] found that the tubercle bacilli contained in pieces of meat which were exposed to air and water and allowed to putrefy, were still virulent after 120 days and when buried in the soil were virulent after 150 days.

That putrefaction, if sufficiently prolonged, lessens the virulence of the bacillus of tuberculosis there can be no doubt. All investigators agree on this. Voelsch[10] found tubercle bacilli present in sputum by straining them after it had been allowed to putrefy for six months, but animals inoculated with this sputum did not develop tuberculosis. The fact that tuberculous sputum is still capable of causing tuberculosis in animals after it has been kept moist and allowed to putrefy for six weeks, shows that the slight putrefaction which occurs under ordinary circumstances before sputum dries, is not sufficient to make the bacillus of tuberculosis inocuous.

As to the influence of *dessication* upon the vitality of the tubercle bacillus, experiments indicate that these germs are not so easily killed by drying as are most of our pathogenic bacteria.

Switzky found that sputum dried on linen lost its virulence after two and one-half to three months, but other observers have found the bacilli active and capable of setting up the disease in guinea pigs after longer periods of drying than this. Sormain found sputum virulent after four months; Schill and Fischer after five and six months; Strauss after six months; Cadiac and Molet found that tuberculous sputum which had been dried and reduced to a powder remained virulent for four and five months. De Toma found tuberculous sputum virulent after ten months when dried at a low temperature (25° C. or below), whereas it loses its virulence in less time if dried at a higher temperature.

Büchner (quoted by Harrington in Practical Hygiene) found the bacilli of tuberculosis present and active in the dust of a room one year after it had been occupied by a consumptive patient. The observations which show the effect of drying for the longest time that I have been able to find, were those of Stone.[12] He allowed sputum to dry for three years, and at the end of that time found the tubercle bacilli not only stainable, but virulent for guinea pigs.

When we stop to consider how soon the sputum dries up and becomes reduced to a powder as it is commonly disposed of by consumptives, it is not surprising that the dust of rooms occupied by tuberculous patients should be a source of danger for months, and even years.

This is true not only in the houses of the ignorant and uncleanly, but also in the homes of the better classes when the sputum is allowed to dry in handkerchiefs. This recalls the experiments of Comet,[13] who found tubercle bacilli present in more than one-third of 147 samples of dust collected from general hospital wards, asylums, and from private houses. Of the guinea pigs which did not die of sepsis, 47 per cent. of those inoculated with the dust collected from seven different hospitals in Berlin developed tuberculosis, while 57 per cent. of those inoculated with the dust taken from the private apartments of consumptives died of tuberculosis.

Perhaps the one agent which has been most trusted by physicians as a natural disinfectant for tuberculous sputum is the influence of *sunlight*. This has doubtless been due to the well-known fact that cultures on artificial media are killed by exposure to sunlight for a short time, and even by diffuse daylight when exposed for a somewhat longer time.

Koch, in 1890, stated before the Tenth International Medical Congress, in Berlin, that tubercle bacilli are killed by an exposure to direct sunlight of from a few minutes to several hours, varying with the thickness of the layer, and that cultures placed near a window are killed in from five to seven days by exposure to diffuse daylight.

From that time to this the medical profession has blindly trusted to light as a natural disinfectant for the sputum, and for the most part the use of more certain germicides has been neglected. I have seen a medical man, himself the victim of tuberculosis, take every precaution to keep the sputum from drying until it could be properly disinfected when he was indoors, but he did not hesitate to spit on the lawn or in the street because of his confidence in the action of strong light.

Migneco,[14] in 1895, was the first to investigate the influence of sunlight upon the *virulence* of the bacillus of tuberculosis. He used a different method, which for practical purposes, is more important than that used by Koch. Instead of relying upon the growth of cultures on artificial media, he spread sputum on linen and exposed this to sunlight for varying periods of time during the hottest season of the year, viz., July, August and September. At the end of different intervals guinea pigs were inoculated, and in this way the virulence was determined. He found that sputum exposed to sunlight continuously for from five to thirty hours still retained its virulence; that the virulence began to be diminished after ten to fifteen hours, depending on the thickness of the layer. The bacilli were unable to withstand exposures of forty hours.

Mitchell and Crouch,[15] of Denver, have made the most valuable experiments on this subject that we have, and their results coincide with those of the earlier experiments of Migneco. They exposed sputum containing almost a pure culture of tubercle bacilli on sterile sand in Petri dishes so as to represent natural conditions when the sputum falls on the soil. The guinea pigs inoculated with sputum exposed in this way from one to twenty-five hours were all tuberculous, while none developed the disease from the injection of sputum exposed for thirty-five hours. Exposures for a longer time entirely destroyed the vitality of the bacilli. They concluded that sputum dried on a sandy soil only begins to lose its pathogenicity after twenty to thirty-five hours exposure to direct sunlight, and is only lost after the last mentioned time. These exposures were made between 10 a. m. and 4 p. m., during September and October, in Denver, Colorado.

The most recent investigations in this line are those of Jousset.[16] From his first experiments (1900) he thought that the virulence of tubercle bacilli was destroyed by exposure to sunlight for from four to seven hours, but hesitated to fix any definite time for sterilization by this method. In a later paper,[17] just published this year (1902), Jousset gives the results of more recent experiments, in which he finds twenty-four hours exposure to direct sunlight insufficient to sterilize sputum, while an exposure of forty-eight hours is sufficient.

It should be understood that in all these experiments the exposures only lasted six or seven hours each day, so that it required four to six consecutive days to destroy the virulence of tubercle bacilli by sunlight. When it is remembered that tuberculous sputum usually dries in four to six hours, it will be seen what opportunity is afforded for the dissemination of the disease, even when nature's most powerful germicide is operating at its best. We have no observations as to how long the bacilli can withstand diffuse daylight and remain virulent, but we know that they may live in the dust of rooms for from one to three years.

The ability to resist putrefaction, drying and light at once suggests that tubercle bacilli may form spores—an idea that has long been entertained by many. The unstained spaces in tubercle bacilli might cause one to think of spores, but these are not refractive in unstained specimens. The recent observations of Swithinbank[18] in showing that these bacilli will grow in cultures and subcultures after exposure to liquid air at a temperature of —186° C. for forty-two days, might strengthen this opinion.

Against this view we have the observations of Bang,[19] of Theobald Smith,[20] and of Russell,[20] each of whom has found that the bacilli of tuberculosis are killed by a temperature of 60° C. for ten minutes, whereas all known spore bearing bacteria are able to resist this temperature. This is of considerable importance in pasteurizing milk for feeding infants.

Whether tubercle bacilli form spores or not, there is abundant proof that they may live and remain virulent for a long time after they are given off from the animal body. As practical sanitarians, we should not rest in a false sense of security, trusting to nature's agencies for the destruction of these germs soon after they leave the animal body, but should take every possible precaution to destroy them by certain, reliable means, and thus prevent their dissemination. If we expect to have the support and co-operation of the people in this matter, medical men must not be passively indifferent, but must take the initiative in combatting this greatest scourge of modern civilization, which Dr. Holmes aptly called "the great white plague."

### BIBLIOGRAPHY.

[1]Reed, Carroll and Agramonte, The Etiology of Yellow Fever, *Phila. Med. Jour.*, October 27, 1900; also *Jour. Am. Med. Assn.*, February 16, 1901; also Experimental Yellow Fever, *Trans. Assn. Am. Phys.*, Vol. XVI, 1901.

[2]Guiteras, *American Medicine*, November 23, 1901.

[3]*Loc. cit.*

[4]Public Health Reports, U. S. Marine Hospital Service.

[5]Reed and Carroll.

[6]Knopf, *Jour. Am. Med. Assn.*, October 30, 1897.

[7]Quoted by Voelsch, *Beiträge zur allgemeinen Pathologie und path. Anatomy*, 1888.

[8]Schill and Fischer, *Mittheil. des Kaiserl. Gesundheitsamtes II*, 553.

[9]Cadéac and Malet, *Lyon Medical*, 1888, LVIII.

[10]*Loc. cit.*

[11]Quoted from Miguel and Cambier, *Bacteriologie*, Paris, 1902.

[12]*Amer. Jour. Med. Sc.*, March, 1891.

[13]Comet, *Zeitschrift f. Hygiene*, Bd. V, 1888, p. 191.

[14]Migneco, *Archiv. für Hygiene*, 1895.

[15]Mitchell and Crouch, *Jour. of Path. and Bact.*, Vol. VI, No. 1, May, 1899.

[16]Jousset, *Comp. Rend. d. l. Soc. d. Biol.*, 1900.

[17]Jousset, *Comp. Rend. d. l. Soc. d. Biol.*, 1902.

[18]Swithinbank, *Proc. British Congress of Tuberculosis*, 1901.

[19]Bang, *Proc. British Congress on Tuberculosis*, 1901.

[20]Quoted by Ravenel, *Bul. Penna. Dept. Agriculture on Tuberculosis in Cattle.*

